# A MEG Study on the Processing of Time and Quantity: Parietal Overlap but Functional Divergence

**DOI:** 10.3389/fpsyg.2019.00139

**Published:** 2019-02-04

**Authors:** Elena Salillas, Milena Korostenskaja, Tara Kleineschay, Shivani Mehta, Alexandra Vega, Eduardo Martinez Castillo

**Affiliations:** ^1^Department of Neurosciences, University of Padova, Padova, Italy; ^2^Functional Brain Mapping and Brain Computer Interface Laboratory, Florida Hospital for Children, Orlando, FL, United States; ^3^MEG Lab, Florida Hospital for Children, Orlando, FL, United States; ^4^Florida Epilepsy Center, Florida Hospital, Orlando, FL, United States

**Keywords:** duration, numerosity, math cognition, parietal cortex, magnetoencephalography (MEG), event related fields (ERFs), source estimation

## Abstract

A common magnitude system for the processing of time and numerosity, supported by areas in the posterior parietal cortex, has been proposed by some authors. The present study aims to investigate possible intersections between the neural processing of non-numerical (time) and numerical magnitudes in the posterior parietal lobe. Using Magnetoencephalography for the comparison of brain source activations during the processing of duration and numerosity contrasts, we demonstrate parietal overlap as well as dissociations between these two dimensions. Within the parietal cortex, the main areas of overlap were bilateral precuneus, bilateral intraparietal sulci, and right supramarginal gyrus. Interestingly, however, these regions did not equivalently correlated with the behavior for the two dimensions: left and right precuneus together with the right supramarginal gyrus accounted functionally for durational judgments, whereas numerosity judgments were accounted by the activation pattern in the right intraparietal sulcus. Present results, indeed, demonstrate an overlap between the neural substrates for processing duration and quantity. However, the functional relevance of parietal overlapping areas for each dimension is not the same. In fact, our data indicates that the same parietal sites rule differently non-numerical and numerical dimensions, as parts of broader networks.

## Introduction

Do our computations of time and quantity share a common cognitive mechanism and a concrete brain overlap? Successful motor behavior might entail the integration between space, time and quantity. According to Bueti and Walsh (2009), these dimensions would share a common analog system, allowing for the contrast between the levels within each dimension: more/less than, bigger/smaller than, faster/slower than. The possibility of a shared common magnitude system has triggered a variety of studies using diverse techniques (e.g., vanMarle and Wynn, 2006; Roitman et al., 2007; Cappelletti et al., 2011; Arend et al., 2014; Dormal et al., 2016; Sokolowski et al., 2017). However, the functional characteristics and neural foundations of this proposed common system are still under debate. Importantly, recent studies (Harvey et al., 2013, 2015) suggest that observed anatomical overlap does not necessarily imply similar neural mechanisms for non-numerical and numerical magnitudes.

The present study will focus on two of these dimensions, numerosity and time, using magnetoencephalography (MEG), which provides a continuous track for neurocognitive processes with good spatiotemporal resolution. MEG differs from other neuroimaging techniques in its temporal millisecond precision. At the same time, it allows for accurate source estimation. Therefore, it should provide information about any neural response to each dimension in time. Such a contribution of MEG to studies in math cognition is essential, as the majority of findings in this field have been mainly based on functional magnetic resonance imaging (fMRI) and stimulation data. Thus, they have provided information on *where* the processes of interest occur without addressing the temporal sequencing of studied functions. The attempts to grasp temporal aspects of numerical and non-numerical processes were made by a fewer number of electroencephalography (EEG) studies, where, in turn, the spatial information is most often missed. Therefore, the use of MEG technique to estimate the source of recorded activity in time is crucial in determining how the activity of different brain areas, involved in processing of both duration and numerosity, unfolds in time.

Neuroimaging evidence so far supports both commonalities and dissociations in the processing of these two dimensions. Interactions are made explicit in the ATOM (“A Theory of Magnitude”) model (Walsh, 2003; Bueti and Walsh, 2009). According to this model, processing of time, space and numbers share in the brain a common magnitude system that allows for the estimation of differences in duration, area or numerosity. The brain locus of this common mechanism would be the parietal cortex, which would integrate these magnitudes in the context of a motor goal (Bueti and Walsh, 2009).

The posterior parietal cortex has been repeatedly shown as a key brain region for *numerical processing* using neuroimaging, implying a pivotal role of the intraparietal sulcus (IPS) (Dehaene et al., 2003; Ansari, 2008; Kaufmann et al., 2011). Parietal areas are, thus, fundamental for the number processing domain. However, they are necessarily complemented by a more extensive network (Arsalidou and Taylor, 2011; Menon, 2015). This network implies prefrontal areas, supporting executive processing; dorsal cingulate, related to working memory (Owen et al., 2005; Arsalidou and Taylor, 2011); the right angular gyrus, involved in spatio-attentional processes (Hillis et al., 2005); as well as the insula, implied by problem solving (Uddin and Menon, 2009). Such an extended network accounts for the bulk of neuroimaging data in the last decades and adds to the involvement of parietal circuits, proposed by the triple code model (Dehaene et al., 2003).

On the other hand, the brain network involved in the processing of *time* is mainly right lateralized. Brain areas, repeatedly shown as relevant for time estimation, are the supplementary motor area and the right prefrontal cortex (Macar et al., 2002; Lewis and Miall, 2006) together with the insula bilaterally. The supplementary motor area is more specifically implicated in the actual processing of time, during the occurrence of the time lapse (Macar et al., 1999; Coull et al., 2008;Morillon et al., 2009). Automatic processing relies on the supplementary motor area and also implies some cerebellar involvement, as well as the contribution of sensoriomotor areas. This system is mainly involved in the continuous measurement of timing, and the controlled processing of time would be dependent on the posterior parietal cortex and dorsolateral prefrontal cortex (Lewis and Miall, 2003b; Rubia and Smith, 2004). Within the parietal cortex, lesion, stimulation and neuroimaging studies demonstrate a clear role of the right angular gyrus and supramarginal gyrus in temporal perception as well (Harrington and Haaland, 1999; Lewis and Miall, 2003b; Wiener et al., 2012). Finally, time perception is consistently altered when impairments in the dopaminergic system are observed in Parkinson’s disease, and a role of precuneus has been clearly related to time processing in these patients (Malapani et al., 2002; Huang et al., 2007a,b; Dormal et al., 2012b).

Thus, brain networks, supporting processing of both time and numerosity, entail a complex system that, more or less pivotally, involves parietal areas. Bueti and Walsh (2009) report associations in the right IPS, based on previous neuroimaging studies. Besides, they illustrate the involvement of the right inferior parietal cortex for time, as well as associations between time and number processing in the dorsolateral prefrontal cortex.

The use of ultra-high field functional MRI, has allowed Harvey and collaborators (Harvey et al., 2013, 2015, 2017) to provide a different approach to the study of a the magnitude parietal system (Walsh, 2003). Indeed, they were able to identify topographic maps for numerical and non-numerical dimensions (size) in the posterior parietal cortex, that are not affected by other non-numerical visual features (Harvey and Dumoulin, 2017). In agreement with the ATOM approach, they confirmed a partial overlap for the processing of size and numerosity. However, contrasting ATOM vision, they showed that the tuning functions for these two dimension differed in the overlapped areas. Besides, the respective responses to ratio did not correlate between dimensions. Tuning widths decreases as preferred numerosity increases, while the opposite occurs for size. These differences in tuning and brain organization led the authors to conclude that the responses, albeit in partly intermingled localizations, arise from different mechanisms. The authors extended their conclusions to other non-numerical dimensions, although the provided empirical data was restricted to size and numerical quantity.

In our current study, we aimed to explore possible associations and dissociations between duration and numerosity with a focus on the parietal lobes, during categorization of durational intervals or the categorization of different numerosities, respectively (Dormal and Pesenti, 2007, 2013; Dormal et al., 2016). In line with Harvey’s proposal (Harvey et al., 2015), we also aimed to complement the debate on the common parietal magnitude system with a meaningful brain-and-behavior link. To that end, we correlated estimated brain sources with behavior. Common activated areas might imply different functions or mechanisms, because they are integrated within a different functional network, together with dissociated areas. Hence, a correlation of brain activity with behavior is important. The common parietal magnitude system proposal (Walsh, 2003; Bueti and Walsh, 2009) seems to assume that a shared parietal loci overlap implies similar functionality for both numerical and non-numerical dimensions, which would imply that those loci activations should show similar correlations with behavior. On the contrary, it is possible to expect that overlapping activations within different networks would give rise to different functionality patterns. This, in turn, should be reflected in correlation differences between the activations of overlapped areas and behavior. In turn, the emergence of different tuned representations in the parietal cortex might be conditioned upon a former inclusion within different functional networks. That is, an extended large-scale network mechanism may be also necessary to give rise to functional specificity and divergence.

## Materials and Methods

### Participants

Ten healthy participants [4 males, 6 females; age (MEAN ± SD): 32.4 ± 10.8 years; 7 right-handed and 3 ambidextrous] were recruited for participation in the current study. The study protocol was approved by the Institutional Review Board of Florida Hospital. Written informed consent to participate in this study was obtained from all participants, in accordance with the Declaration of Helsinki. All subjects underwent a health screening questionnaire to identify physical or mental health problems, as well as MRI and MEG questionnaires to identify the presence of metal in their body that would exclude them from participation in this study. The handedness of the participants was determined by using Edinburgh Handedness Inventory (Oldfield, 1971).

### Procedure and Stimuli

All study participants performed two tasks:

(1) Numerical magnitude categorization (referred here to as *Numerosity Categorization task*); and
(2) Non-numerical magnitude categorization of duration (referred here to as *Duration Categorization task*).

**FIGURE 1:**
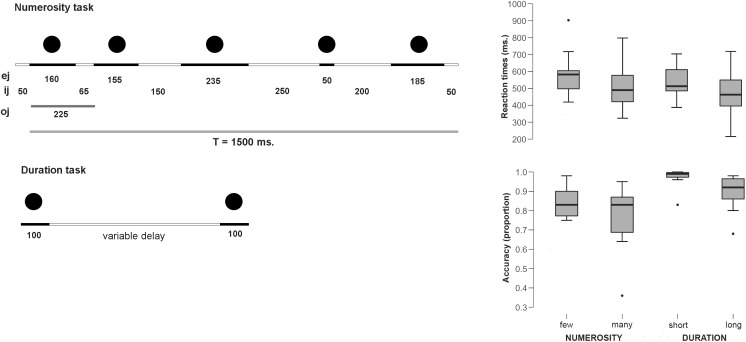
Experimental paradigms and behavioral results. **(Left)** Scheme for the sequential presentation for the numerosity task. Total duration was kept constant and temporal ratio, rhythm biases and pattern recognition were controlled. The figure provides an example of non-periodic series with five dots and a total duration of 1500 ms (*T* = total signal duration; *o*_j_ = dot onset duration; *e*_j_ = dot duration; *i*_j_ = interdot duration) (adapted from Dormal et al., 2006). **(Right)** Behavioral results (RTs and Accuracy proportions).

In the *Numerosity Categorization task*, a set of central flashing dots had to be classified as *few* or *many*. The participants responded with different button press to one or another choice. The details of the paradigm were as follows: First, a fixation cross was presented in the middle of the screen for 200 ms and a series of 5, 6 (few), 8 or 9 (many) flashed dots were presented. Each numerosity comprised 35 trials for a total of 70 trials per category. All series were composed of gray dots (diameter: 3.5 cm) and were constructed using non-periodic signals so that temporal ratios did not constitute a potential confounding variable as well as the rhythm biases and pattern recognition were avoided (as in Dormal and Pesenti, 2007, 2013; Dormal et al., 2016). Although the time between the presentation of the dots was variable, the duration of the presented sequence stayed always the same independently from the number of presented dots and was equal 1500 ms. This rapid presentation and the use of numerosities above the subitizing range avoided counting during the task (see method in Dormal et al., 2006). The durations of each dot (*e*_j_) and the inter-dot intervals (*i*_j_) (see Figure 1) were both variable (*e*_j_ between 50 and 235 ms and *i*_j_ between 50 and 250 ms); to avoid pattern recognition, each series involved at least one *e*_j_ and one *i*_j_ of 50 ms and one longer than 200 ms, and each series began and finished with an *i*_j_ of 50 ms. The ratio deviation score (DS ratio) provides a measure of deviation in terms of the *o*_j_/T (total duration) ratio, and a pattern deviation score (DS pattern) provides a measure of deviation in terms of the signal patterns. The DS ratio was kept constant for all stimuli and equal to 216 ms. The DS pattern was always kept above 160 (see Dormal and Pesenti, 2007, 2013; Dormal et al., 2016). After disappearance of the last dot, a blank screen was presented to the participant for 950 ms, during which the participant was instructed to make his response on whether the trial belonged to *few* or *many* category.

In the *Duration Categorization task*, participants were asked to decide whether the elapsed time between a pair of centrally presented dots was *long* or *short*. The participants responded with different button press to one or another choice. The paradigm was executed as follows: first, a fixation cross was presented in the middle of the screen for 200 ms. Afterwards, a central dot #1 was presented on the screen for 100 ms, followed by 100 ms presentation of a dot #2 with two different presentation intervals: 200 or 300 ms (*short* interval) and 500 or 600 ms (*long* interval). Each length comprised 70 trials. After the last dot had disappeared, a blank screen was presented to the participant for 950 ms. During this time, the participant was prompted to make a choice and press a button, indicating whether the interval between circle #1 and #2 was *short* or *long*. This was followed by 1000 ms interval, after which the next trial begun. Participants were presented with series of familiarization trials, until they understood the tasks and were able to perform them well.

### Data Acquisition

#### Structural Data (MRI) Acquisition

Using a 3T MRI (Philips 3T Achieva), a multiplanar T1-weighted image without contrast was acquired for each study participant. The T1 sequence used the Turbo Field Echo (TFE) 3D acquisition protocol with a voxel size of 1.0 mm, for 160 continuous axial slices. FOV 240 × 240 and TR/TE = 9.8/4.6 ms. The total scan time was 4:23 min.

#### Functional Data (MEG) Acquisition

We acquired magnetic brain signals using a whole-head MEG system with 306 sensors (204 planar gradiometers, 102 magnetometers, Elekta Neuromag TRIUX, Elekta, Stockholm, Sweden). Participants were placed in the supine position into the scanner located inside the magnetically shielded room (Vacuumschmelze GmbH, Hanau, Germany). During acquisition, the MEG data was sampled at 1000 Hz and the head position of each study participant was recorded, before and after each task, relatively to the MEG sensors by using five head-localization coils. The head-localization coil positions and scalp outline (roughly 300 points) were digitized using a three-dimensional digitizer (Fastrak; Polhemus, Colchester, VT, United States). All digitized points were used to achieve accurate co-registration between the structural T1 image of the participant and the position of the head inside the MEG helmet with the sensors.

The stimuli were presented via projector onto the 80 cm screen, positioned on the ceiling above the participant at a distance of approximately 200 cm. The stimuli presentation paradigms were programmed by using STIM2 (Neuroscan, Compumedics) software. At the time when the events of interest have occurred, a trigger was sent to mark the trigger channel within the MEG recording.

### Data Processing

Artifacts were removed from the recorded MEG data by spatially filtering the raw data offline, using the temporal extension of Signal Space Separation (tSSS), as implemented in the MaxFilter software (Elekta Neuromag version 2.1). Details and parameter settings of this approach have been described elsewhere (Taulu and Simola, 2006).

Following the described preprocessing (tSSS), the data was imported into the Brainstorm software^1^, where a SSH correction for blinks and cardiac artifacts was performed, following visual inspection of topographical maps and the selection of predominant sensors, associated with each artifact type.

#### Sensor Space

##### Event related fields (ERFs)

After the MEG signal pre-processing, the corrected signal was segmented with the pre-stimulus baseline of 200 ms and the time window of 1400 ms after the event. Once segmented, the events of interest were averaged for each condition. The source of neuromagnetic activity was estimated from the sensor averages. The ERFs analyses were focused on five different clusters of gradiometers in each hemisphere, allowing for the homogenization of sensor location across participants: Frontal, Paracentral, Temporal, Parietal, and Occipital. The parietal clusters were further divided into inferior, superior, and posterior.

Contrasts were performed by comparing each of the conditions for each magnitude dimension. For quantity, the condition containing more dots (*many*) was contrasted with the condition containing fewer dots (*few*). For duration, the condition of longer duration (*long*) was contrasted with the condition of shorter duration (*short*). In both cases, the ERFs were measured from the beginning of the last stimulus, that is, from the second dot in the duration block and from the last dot in the sequence in the quantity block. Notably, this segmentation implies the measure of the processes *after* the appearance of the last stimulus, thus a response to the same visual stimulus in each condition, preceded by the computation of duration, or numerosity. The timing of the ERFs components established the temporal latency of interest for source time analysis, restricting the 1400 possible brain source configurations to delimited and averaged time windows of interest.

#### Source Space

##### Source analysis (MNE)

The method used to estimate the dipoles distributed in the cortex was Minimum Norm Estimates (M. Hämäläinen, software MNE). The cortical surface geometry from the structural MRI was obtained by using the Freesurfer software. The reconstructed surface was automatically reduced to a value close to 15000 vertices (depending on the participants’ anatomy) to facilitate the analysis. So, there were about 15000 potential 1-vertice sources, 7500 by each hemisphere. These cortical surfaces were aligned with the sensors using the interface provided by Brainstorm software. Before calculating the MNE solution for each participant and condition average, a covariance matrix, based on the recording, was obtained with respect to the baseline. An overlapping spheres method was used to estimate the forward model. The MNE solution was then calculated for each millisecond (-200 to 1400 ms) on the cortical surface. The source maps were then normalized (*Z*-score minus baseline) and projected to a cortical template based on a default anatomy (ICBM152), to allow for a group analyses.

#### Data Analysis

The behavioral data (accuracy and RTs) were tested for normality (Shapiro–Wilk *W*) and normal distributions (RTs) were then compared through *t*-tests (many vs. few for numerosity; long vs. short for duration). For non-normal distributions (accuracy), the Mann–Whitney *U* test was applied to contrast each of the dimensions.

For the ERFs data, averaged ERFs for each dimension were included into a Cluster (5: Frontal, Paracentral, Temporal, Parietal, and Occipital) × Hemisphere (2: left, right) × Numerosity or Duration (2: few, many or 2: short, long) ANOVA. A second ANOVA was performed on the parietal sub-clusters: Cluster (3: inferior, superior, posterior) × Hemisphere (2: left, right) × Numerosity or Duration. Given the large numbers of sensors (20 locations × 2 gradiometers) behind the clusters, 2 (Hemisphere) × 2 (Duration or Numerosity) ANOVAs were also performed for each cluster including hemisphere in the design. We focused on the significant effects and interactions involving Numerosity or Duration, as well as on asymmetric amplitudes for one of the values in each dimension, assuming that each dimension was differently processed by each hemisphere. Those effects were crucial for guiding the subsequent source estimation analyses. We finally computed Holm–B&H corrected one-tailed *t*-tests (short minus long/few minus many/left hemisphere minus right hemisphere), to observe the directionality of amplitude differences. *Post hoc* power analyses were performed at the cluster level, in order to estimate the achieved power.

For the source data, absolute values of the source were taken for statistical group analysis. A first whole brain analysis was followed by an analysis restricted to the parietal cortex. The different conditions were contrasted through paired F-tests (many vs. few for numerosity and long vs. short for duration) using SPM8 (The FIL Methods Group) for each of the time windows derived from the ERFs.

Finally, Pearson’s r correlation coefficients were obtained between the absolute difference in RTs or accuracy (many vs. few or long vs. short) and the absolute difference in source values for each dimension (many vs. few or long vs. short), time window, and parietal site of interest.

## Results

### Behavioral Data

Average reaction times for the *Numerosity Categorization* task were 589.7 ms (*SE* 43.9) for the *few* condition and 514.4 ms (*SE* 48.97) for the *many* condition. Thus, participants were faster in judging when the series contained a larger amount of dots than the smaller ones [*t*(9) = 2.44; *p* = 0.037; *d* = 0.77]. None of the RTs distributions deviated from normality (few: *W* = 0.89; *p* = 0.17; long: *W* = 0.94; *p* = 0.59). Participants tended to be more accurate for *few* dots (proportion = 0.85; *SE* 0.02) than for *many* dots responses (proportion = 0.77; *SE* 0.05), although this difference did not reach statistical significance [*U* = 190.5 n.s. (critical U for *p* < 0.05: 138)].

Average reaction times for the *Duration Categorization* task were 540 ms (*SE* 31.2) for the *short* condition and 475.3 (*SE* 46.45) for the *long* condition. This difference was significant, with faster responses for the *long* condition [*t*(9) = 2.3; *p* = 0.04; Cohen’s *d* = 0.75]. None of the RTs distributions deviated from normality (short: *W* = 0.95; *p* = 0.72; long: *W* = 0.98; *p* = 0.97). Participants tended to be more accurate for the *short* condition (proportion = 0.97; *SE* 0.016) than for the *long* condition (proportion = 0.89; *SE* 0.03), although once again, this difference did not reach statistical significance [*U* = 193.5 n.s. (critical *U* for *p* < 0.05: 138)].

Only epochs corresponding to correct responses were used for the following MEG analyses at both sensor and source levels.

### MEG: Sensor Space

**FIGURE 2:**
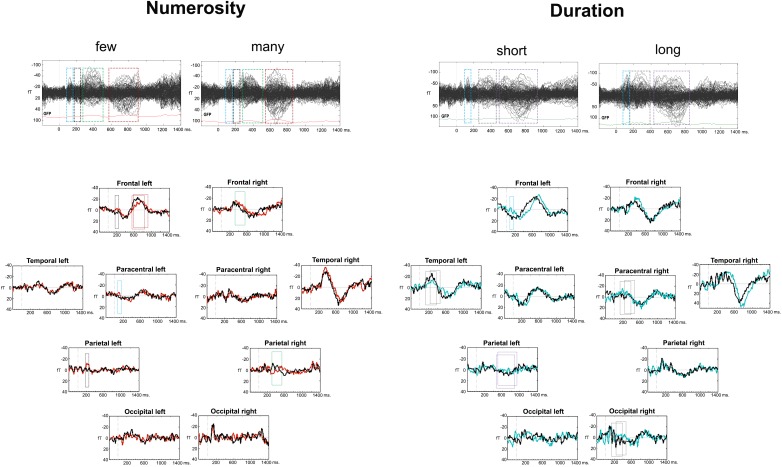
Event related fields (ERFs) for the two dimensions. The upper part of the figure contains a butterfly view off all the gradiometers for each condition and dimension. The bottom part includes the cluster averages for the two dimensions with conditions overlapped. The four or three consecutive latencies bands for analyses are displayed in squares, for numerosity and duration, respectively.

A visual analysis of the Event Related Fields (ERFs) in each of the dimensions (*duration* and *numerosity*) and for each of the dimension values (*long*-*short* and *many*-*few*) showed a similar sequence of components after the last stimulus, albeit with different latencies for the two dimensions. This was an expected finding because of two reasons: first, the ERFs were measured after the same visual stimulus in both tasks: the last dot of the series for numerosity and the second dot for duration; second, the tasks were also comparable between each other, as they both were a categorization task. Figure 2 depicts the timing of ERF components, based on the deflections of all gradiometers, as well as on gradiometers clusters. For *numerosity*, the first component arose between 80 and 160 ms after the stimulus, followed, in some clusters, by a second early component between 170 and 250 ms. Two later components were found subsequently: at 240–520 ms and a last component with different latencies for each condition, starting slightly earlier for the *many* condition (550–850 ms) than for the *few* condition (590–940 ms). For *duration*, a similar time-wise early component arose between 80 and 160 ms. This component was followed by two later components with earlier latencies for the *long* condition: (*long*: 160–400 ms vs. *short*: 240–460 ms and *long*: 420–850 ms vs. *short* 490–950 ms).

#### Numerosity

During the 80–160 ms ERF component, the left and right Paracentral clusters differed in the processing of the *many* condition [*F*(1,9) = 9.94; *p* = 0.012; η^2^= 0.52], with larger amplitude in the left hemisphere [*t*(9) = 3.15; *p* = 0.006; *d* = 0.99; Holm–B&H corrected hereafter]. The Left Paracentral cluster showed larger negativity for the *few* condition [*t*(9) = -1.97; *p* = 0.04; *d* = 0.62]. In the 170–250 ms time frame, a numerosity × hemisphere interaction was found in the Frontal cluster [*F*(1,9) = 5.922; *p* = 0.038; η^2^ = 0.39]. There were significant differences between the two numerosity values in the Left Frontal cluster [*F*(1,9) = 6.87; *p* = 0.028; η^2^ = 0.43] with larger negativities for the *few* condition [*t*(9) = -2.62; *p* = 0.01; *d* = 0.83]. In the Parietal cluster, a trend for a numerosity effect was shown [*F*(1,9) = 3.4; *p* = 0.097; η^2^= 0.27] as being originated by a significant numerosity effect in the Left Posterior Parietal cluster [*F*(1,9) = 5.52; *p* = 0.04; η^2^ = 0.38], with larger amplitude for the *few* condition [*t*(9) = -2.65; *p* = 0.013; *d* = 0.84]. The later ERF component (240–520 ms) showed a cluster × numerosity interaction effect [*F*(4,36) = 4.67; *p* < 0.001; η^2^= 0.53]. A significant contrast was localized frontally in the right hemisphere [*F*(1,9) = 7.89; *p* = 0.02; η^2^ = 0.47] with larger amplitudes for the *few* condition [*t*(9) = -2.81; *p* = 0.02; *d* = 0.89]. The right parietal sub-clusters showed a numerosity effect inferiorly [*F*(1,9) = 9.918; *p* = 0.012; η^2^ = 0.52] with larger amplitudes for the *many* condition [*t*(9) = 2.03; *p* = 0.04; *d* = 0.64]. Finally, a cluster × numerosity interaction was found in the latest window (*few*: 590–940 ms; *many*: 550–850 ms) [*F*(4,36) = 3.42; *p* = 0.02; η^2^ = 0.27] – again, localized frontally [numerosity × hemisphere interaction: *F*(1,9) = 5.22; *p* = 0.048; η^2^ = 0.37], in the left hemisphere [Left Frontal cluster: *F*(1,9) = 7.11; *p* = 0.026; η^2^ = 0.44] with larger negativity for the *many* condition [*t*(9) = 2.67; *p* = 0.013; *d* = 0.84]. *Post hoc* power analyses at the cluster level revealed an achieved power(1-β) between 0.65 (based on effect size *f* = 0.42) and 0.99 (based on effect size *f* = 0.68) with all achieved power values higher than 0.93, except for the reported minimum (0.65).

#### Duration

During the appearance of the 80–160 ms ERF component, the left Frontal cluster showed a trend for Hemisphere × Duration interaction: [*F*(1,9) = 3.96; *p* = 0.078; η^2^ = 0.31], due to a duration effect localized in the left hemisphere [*F*(1,9) = 22.06; *p* = 0.001; η^2^ = 0.71] with larger negativities for the *short* condition [*t*(9) = -4.7; *p* < 0.001; *d* = 1.48]. The *long* condition was also less negative in the left hemisphere than in the right hemisphere [*F*(1,9) = 10.79; *p* = 0.009; η^2^ = 0.55; *t*(9) = 3.28; *p* = 0.005; *d* = 1.04]. In the following components (*long*: 160–400 ms; *short*: 240–460 ms), a hemisphere × cluster × duration interaction was found [*F*(4,36) = 3.11; *p* = 0.03; η^2^= 0.26]. Clusters, yielding significant differences, were the following: the Paracentral cluster [*F*(1,9) = 5.33; *p* = 0.046; η^2^= 0.37], where the right hemisphere showed a stronger effect of duration, with larger negativities for the *short* duration [*t*(9) = -1.86; *p* = 0.048; *d* = 0.58] and the Left Temporal cluster [*F*(1,9) = 10.22; *p* = 0.011; η^2^ = 0.53], with larger negativity for the *long* condition [*t*(9) = 3.19, *p* = 0.005, *d* = 1.01]. In the last evaluated ERF component (*long*: 420–850 ms; *short*: 490–950 ms), the left inferior parietal cluster showed an effect of duration [*F*(1,9) = 7.48; *p* = 0.023; η^2^ = 0.45] with larger positive amplitudes for the *long* condition [*t*(9) = -2.067; *p* = 0.034; *d* = 0.65]. *Post hoc* power analyses on the cluster level revealed an achieved power (1-β) between 0.63 (based on *f* = 0.33) and 0.99 (based on an *f* = 0.65). All power values were higher than 0.98, except for the reported minimum (0.63).

### MEG: Source Space

**FIGURE 3:**
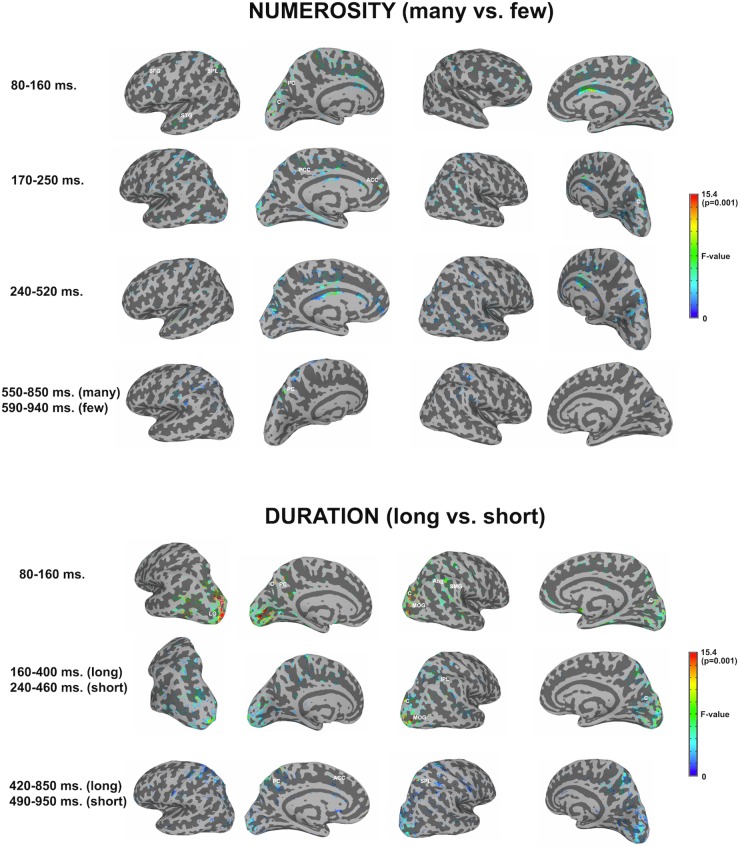
Whole brain analyses for the two dimensions. The image depicts the *F*-test values from the many minus few contrast (numerosity) and from long minus short contrast (duration). *p* < 0.01; >10 voxels. SFG, superior frontal gyrus; SPL, superior parietal lobule; STG, superior temporal gyrus; PC, precuneus; C, cuneus; PCC, posterior cingulate cortex; ACC, anterior cingulate cortex; LG, lingual gyrus; MOG, middle occipital gyrus; Ang, angular gyrus; SMG, supramarginal gyrus; IPL, inferior parietal lobule.

Figure 3 shows significant differences in the source space for the two dimensions (*F*-test contrasts *long* vs. *short* or *F*-test contrasts *many* vs. *few*) across the different windows. Tables 1, 2 show significant clusters (cluster size > 20 voxels; uncorrected *p* < 0.001) for numerosity and duration, respectively.

**Table 1:** Numerosity: whole brain analyses.

		Cluster size (*F*-value)		MNI coordinates					
			
		LH	RH	LH			RH		
**(A) Numerosity 80–160 ms**									

Middle Frontal Gyrus	BA 6		43(10.86)				27	27	57
Paracentral Lobule	BA 31	23(12.26)		-9	-8	47			
Subcallosal Gyrus	BA 34		21(9.25)				19	6	-21
**Superior Frontal Gyrus**	BA 6		20(11.22)				26	38	54
	**BA 8**	**218(18.67)**		-**25**	**32**	**51**			
Cingulate Gyrus	BA 24	20(11.64)		-7	-12	42			
**Cuneus**	BA 18		45(11.31)				20	-101	15
	**BA 18**	**115(15.96)**	25(13.44)	-**12**	-**88**	**31**	5	-89	24
	**BA 7**	**43(22.26)**		-**14**	-**75**	**41**			
	BA 19	22(8.83)		-9	-78	38			
Fusiform Gyrus	BA 37		26(14.26)				31	-53	-8
Middle Occipital Gyrus	BA 18		41(15.12)				12	-98	21
Inferior Parietal Lobule	BA 39	20(10.98)		-35	-60	49			
**Superior Parietal Lobule**	**BA 7**	**32(15.26)**		-**31**	-**63**	**62**			
Postcentral Gyrus	BA 3		95(12.22)				41	-19	49
	BA 3		44(10.94)				16	-30	71
**Precuneus**	**BA 19**	**210(22.30)**		-**21**	-**81**	**51**			
	**BA 7**	**114(15.67)**		**0**	-**71**	**51**			
Inferior Temporal Gyrus	BA 37	27(13.22)		-63	-56	-6			
Middle Temporal Gyrus	BA 21		40(14.75)				72	-15	-10
Superior Temporal Gyrus	BA 22	156(14.44)		-67	-10	1			

**(B) Numerosity 170–250 ms**									

Inferior Frontal Gyrus	BA 9	21(10.06)		-49	9	27			
**Anterior Cingulate**	**BA 32**	**28(19.23)**		-**9**	**46**	**3**			
**Posterior Cingulate**	**BA 4**	**21(16.02)**		-**1**	-**39**	**47**			
**Cuneus**	BA 18	81(14.76)		-9	-76	36			
	**BA 19**		**81(22.35)**				**14**	-**93**	**35**
Superior Parietal Lobule	BA 7		11(9.91)	-22	-41	62			
Middle Temporal Gyrus	BA 21	27(12.07)		-68	-8	-12			

**(C) Numerosity 240–520 ms**									

		**Cluster size (*F*-value)**					**MNI coordinates**		
				
		**RH**					**RH**		

Precuneus - Cuneus	BA 19	32(12.18)					22	-89	39

**(D) Numerosity 550–940 ms**									

		**Cluster size (*F*-value)**					**MNI coordinates**		
				
		**LH**					**LH**		

Precuneus	BA 19	57(11.52)					-9	-78	50


*Uncorrected p < 0.01; ***p < 0.001 highlighted***.*

*Cortical clusters of a minimum of 20 voxels and peak F-values with p ≤ 0.01 for the many vs. few contrast. Clusters with peak F-values with p ≤ 0.001 are highlighted.*

**Table 2:** Duration: whole brain analyses.

		Cluster size (*F*-value)		MNI coordinates					
			
		LH	RH	LH			RH		
**(A) Duration 80–160 ms**									

Middle Frontal Gyrus	BA 6	51(9.92)	44(11.25)	-27	18	54	53	9	39
Precentral Gyrus	BA 6	82(12.93)		-36	-3	61			
Anterior Cingulate	BA 25		38(9.73)				5	6	-9
Cingulate Gyrus	BA 24		39(12.2)				7	9	43
**Posterior Cingulate**	**BA 30**	**47(18.37)**		-**18**	-**60**	**16**			
**Cuneus**	**BA 18**		**46(19.91)**				**19**	-**86**	**25**
	**BA 19**	**291(19.65)**		-**1**	-**79**	**42**			
**Lingual Gyrus**	**BA 17**	**2163(38.84)**		-**15**	-**105**	-**3**			
**Middle Occipital Gyrus**	**BA 18**		**498(28.03)**				**32**	-**86**	**4**
**Angular Gyrus**	**BA 39**		**22(19.28)**				**39**	-**60**	**42**
**Supramarginal Gyrus**	**BA 40**		**87(16.29)**				**53**	-**45**	**44**
Postcentral Gyrus	BA 40		88(13.03)				69	-25	18
**Precuneus**	**BA 19**	**21(15.6)**		-**30**	-**82**	**44**			
	**BA 31**	**92(17.46)**	79(13.93)	**0**	-**65**	**29**	6	-72	30
	**BA 7**	**60(24.24)**		-**2**	-**61**	**41**			
Insula	BA 13		211(14.73)				49	-26	14
Fusiform Gyrus	BA 19	37(10.8)		-50	-70	-11			
Superior Temporal Gyrus	BA 22	36(12.88)		-47	-58	18			

**(B) Duration 160–460 ms**									

Cuneus	BA 18		40(11.05)				19	-103	12
**Cuneus**	**BA 18**		**56(22.58)**				**5**	-**79**	**22**
Fusiform Gyrus	BA 18		28(11.8)				32	-98	-8
Lingual Gyrus	BA 18	89(13.99)		-14	-106	2			
**Middle Occipital Gyrus**	**BA 18**		**655(36.4)**				**32**	-**86**	**6**
**Inferior Parietal Lobule**	**BA 39**		**24(18.22)**				**40**	-**61**	**44**
Precuneus	BA 7	39(11.38)		-10	-46	58			
Supramarginal Gyrus	BA 40	24(13.01)		-61	-51	44			

**(C) Duration 420–950 ms**									

**Cingulate Gyrus**	**BA 32**	**63(16.21)**		-**1**	**22**	**37**			
**Cuneus**	**BA 19**	**97(16.69)**	23(14.08)	-**15**	-**81**	**45**	20	-80	42
Postcentral Gyrus	BA 3	29(11.7)		-34	-27	60			
Postcentral Gyrus	BA 5	63(11.26)		-42	-38	66			
Precuneus	BA 19	22(11.17)		-26	-79	43			
**Precuneus**	**BA 7**	**44(14.6)**		-**1**	-**61**	**38**			
**Superior Parietal Lobule**	**BA 7**	55(12.25)	**106(28.61)**	-6	-60	62	**23**	-**63**	**64**
Superior Temporal Gyrus	BA 22	22(13.41)		-61	2	-9			


*Uncorrected p < 0.01;****p < 0.001 highlighted.***

*Cortical clusters of a minimum of 20 voxels and peak F-values with p ≤ 0.01 for the long vs. short contrast. Clusters with peak F-values with p ≤ 0.001 are highlighted.*

#### Numerosity

For numerosity, apart from initial occipital differences (cuneus, middle occipital gyrus), the effects started at the first studied latency band (80–160 ms) at the left superior frontal gyrus and the left precuneus. Both middle and superior temporal gyri showed effects in this latency band. Effects were very weak in the successive windows. In the second early latency band (170–250 ms), the highest difference was found in the left anterior cingulate and right cuneus.

#### Duration

For duration, differences at occipital sites were evident for the first latency band (80–160 ms): left lingual gyrus, right middle occipital gyrus or left and right cuneus. Regarding parietal areas, the highest difference appeared in the left precuneus and in the right angular gyrus as well as in the supramarginal gyrus. Bilateral occipital differences in source activation remained less evident in the second latency band (*long*: 160–400 ms; *short:* 240–460 ms). Parietal effects were found in the right inferior parietal lobule and right intraparietal sulcus. In the last explored latency band (*long*: 420–850 ms; *short*: 490–950 ms), the effects were localized in the left cuneus, the anterior part of the cingulate gyrus, in the left precuneus and in the right superior parietal lobule.

#### Focus on Parietal Lobes

**FIGURE 4:**
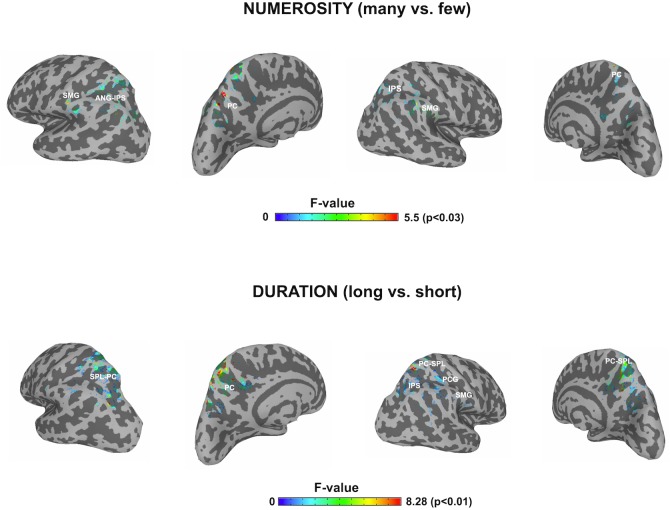
F contrasts after parietal masks for the two dimensions. The picture depicts any difference between the respective conditions for each dimension restricted to the parietal lobes, after collapsing the three windows of analyses. SMG, supramarginal gyrus; ANG, angular gyrus; IPS, intraparietal sulcus; PC, precuneus; SPL, superior parietal lobule; PCG, precentral gyrus.

In order to contrast the parietal effects between the two dimensions as our main objective, the *F* contrasts were performed for each of the windows and for each dimension by using a parietal mask and the effects of all windows were collapsed for each dimension. This outlined *any* parietal involvement along the three temporal windows for each dimension. Thanks to the temporal precision of the MEG technique, a sensitive detection of parietal areas, involved in the processing of each studied dimension, could be obtained. Figure 4 shows the cross-latencies overlap for each dimension. Table 3 shows significant parietal clusters (cluster size >20 voxels) for each time interval.

**Table 3:** Parietal mask analyses.

NUMEROSITY	LH							RH						
		
			Cluster size	*F*	MNI coordinates					Cluster size	*F*	MNI coordinates		
**80–160 ms**	**IPS-AG**	**BA 39**	96	10.98	-35	-60	49	**SMG**	**BA 40**	21	9.31	58	-36	48
	**Precuneus**	**BA 19**	27	8.30	-25	-80	48			34	9.18	64	-51	42
		**BA 7**	94	9.36	-25	-71	50	**SPL-Precuneus**	**BA 7**	71	18.36	11	-62	70
			251	15.67	0	-71	51							
**170–250 ms**	**SMG**	**BA 2**	49	7.80	-60	-24	39	**SMG**	**BA 40**	24	8.39	59	-45	31
**240–520 ms**	**SMG**	**BA 40**	29	6.46	-56	-30	45	**SMG**	**BA 40**	23	8.85	52	-26	48
	**Precuneus**	**BA 7**	69	7.38	-1	-66	57							
**550–940 ms**	**Precuneus**	**BA 19**	110	11.52	-9	-78	50							

**DURATION**	**LH**							**RH**						
		
			**Cluster size**	***F***	**MNI coordinates**					**Cluster size**	***F***	**MNI coordinates**		

**80–160 ms**	**Precuneus-Cingulate**	**BA 31**	79	17.46	0	-65	29	**IPS**	**BA 39**	22	19.28	39	-60	42
	**Precuneus**	**BA 7**	60	24.24	-2	-61	41	**SMG**	**BA 40**	87	16.29	53	-45	44
**160–460 ms**	**Precuneus**	**BA 7**	28	11.38	-10	-46	58	**IPS**	**BA 39**	24	18.22	40	-61	44
**420–950 ms**	**Precuneus**	**BA 7**	44	14.60	-1	-61	38	**SPL-Precuneus**	**BA 7**	49	11.03	9	-63	65
			55	13.14	-6	-75	56							
	**Precuneus**	**BA 19**	22	11.17	-26	-79	43							
	**SPL-Precuneus**	**BA 7**	26	13.73	-25	-79	48							


*Cortical clusters after applying a posterior parietal cortex mask of a minimum of 20 voxels and peak F-values with p ≤ 0.03 for the many vs. few contrast and p ≤ 0.01 for the long vs. short contrast.*

This focus on parietal areas for *numerosity* yielded significant source differences in the left angular gyrus/left intraparietal sulcus, precuneus for the first window, as well as in the right supramarginal gyrus and right superior parietal lobule. In a second early window, the bilateral inferior parietal activation continued. In the following window, inferior parietal lobules showed the effect bilaterally, together with left precuneus. Only precuneus showed an effect in the last window.

For *duration*, the parietal areas with observed significant effects were initially left precuneus, right intraparietal sulcus and right inferior parietal areas. During the second latency band, the effect appeared at left precuneus and right intraparietal sulcus, with left precuneus remaining responsive, together with right superior parietal lobule in the last window.

**FIGURE 5:**
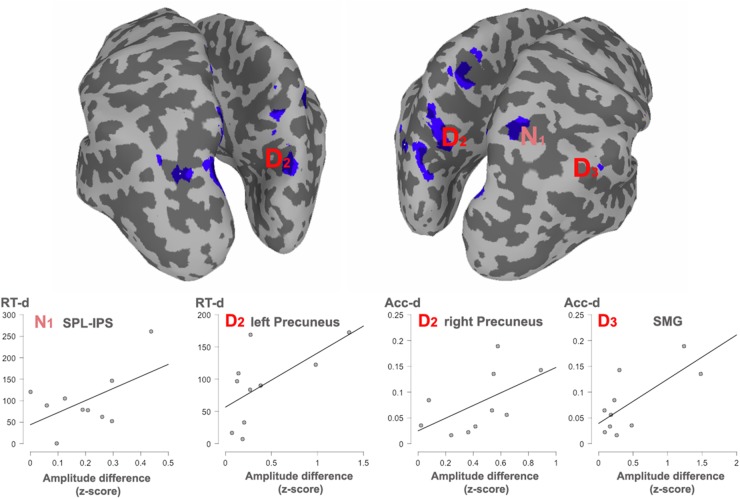
Significant correlations with behavioral effects, between all the clusters showing overlap. The cortex shows an overlaid mask with the overlapped areas between dimensions. The mask was smoothed for visualization. Areas showing a significant correlation with reaction times (RT) or accuracy (Acc) are indexed. N_1_, Numerosity (60–180 ms); D_2_, Duration (long: 160–400 ms; short: 240–460 ms). D_3_, Duration (long: 420–850 ms; short: 490–950 ms).

Afterwards, the *two dimensions* were overlapped and the over-threshold common regions were extracted. Table 4 and Figure 5 show the clusters of overlap across dimensions within the parietal cortex. The largest overlap was found in different sections of the left precuneus. Common regions were also found in the superior parietal lobules bilaterally, including the intraparietal sulcus. Finally, a small region was shown in the right supramarginal gyrus as well as in the right precuneus.

**Table 4:** Clusters of overlap in the parietal lobe.

		BA	Cluster size	MNI coordinates			Behavior	
					
							N	D
**RH**								
	**Supramarginal**	BA40	11	65	-50	40		A
	**Gyrus**
	**SPL- IPS**		10	24	-62	60	RT	
	**Precuneus**	BA19	18	18	-80	38		A
**LH**								
	**Precuneus**	BA7	182	-5	-76	36		RT
			86	-16	-81	43		
			10	-6	-59	70		
		BA5	84	-7	-44	55		
	**SPL-IPS**		41	-26	-80	46		


*Two masks with over threshold activations for each dimension were overlapped and common areas extracted. The table displays clusters from this overlap and corresponds to the overlay in Figure 4. RT, Correlated to Reaction Times; A, Correlated to Accuracy*.

#### Correlation Between Behavior and Brain Activity

In order to observe the functional significance of the areas with overlapped activation between the two dimensions, the absolute difference in source activity between *long* and *short* conditions and between *many* and *few* conditions were correlated with the absolute differences in reaction times (RTs) and accuracy proportion for *long* and *short* conditions as well as for *many* and *few* conditions in the duration and quantity tasks, respectively. This analysis was done for each of the temporal windows in each dimension. Given that negative modulations of behavioral responses have less functional meaning, only positive correlations were considered, that is, when larger differences at the behavioral level were positively correlated with larger differences at the source level across participants. In turn, those correlations indicated that the modulation in source activity was directly related to the modulations in behavior. Figure 4 shows significant correlations.

For *numerosity*, in the 80–160 ms window, the observed differences in the RTs were positively correlated with the activity in the right superior parietal lobule (IPS, coordinates 24, -62, 60; *r* = 0.53; *p* = 0.057). This correlation continued in the second early temporal window 170–250 ms: (*r* = 0.51; *p* = 0.06). No correlational effects were found in the other two windows.

For *duration*, the activity difference within the left precuneus positively correlated with the RTs differences (BA7, coordinates -5, -76, 36; *r* = 0.61; *p* = 0.03) in the second temporal window from 160 to 460 ms. Moreover, in this time window, the difference in activity within the right precuneus positively correlated with accuracy differences (BA19, coordinates 18, -80, 38; *r* = 0.55; *p* = 0.04). In the last temporal window, the difference in activity within the right supramarginal gyrus positively correlated with the RT accuracy differences (BA40, coordinates 65, -50, 40); *r* = 0.72; *p* = 0.009).

## Discussion

### Behavioral Effects

Both studied dimensions (non-numerical and numerical) yielded faster responses for larger values (*many, long*) and a trend for higher accuracy for the lowest values (*few, short*). Shorter reaction times for larger magnitudes have been reported elsewhere (Karolis and Butterworth, 2016), most likely indicating that their associated perceptual mechanism has more information available for making the decision. These effects will acquire further meaning when correlated to the brain activity and, in turn, they show that variations in the dimensions produced differences at the behavioral level as well.

### Overall Sensor and Source Effects for Each Dimension

During the estimation of *numerosity*, the ERF effects appeared in paracentral, parietal, and frontal sites, similarly to what was observed in the source space analyses. Most of the effects for quantity processing at the source level were solely restricted to a very early moment in time, between 80 and 160 ms. The brain sources that maximally accounted for the differences in quantity estimation, were located in the left precuneus. These parietal areas were complemented by the left superior parietal and superior frontal sources. After this early activity, the left anterior cingulate completed the processing.

During the estimation of *duration*, frontal, paracentral and parietal sensors, showed effects throughout the three windows of analysis. These effects appeared to be complemented by the left temporal cluster. However, given the number of sensors included within the clusters, these ERF differences could reflect the effects originated either in the temporal or the parietal brain areas. At the source space level, the left precuneus consistently showed activity throughout the three temporal windows of analysis, being complemented by the left anterior cingulate during the last window. In the right hemisphere, the areas sensitive to the processing of differences in duration were mainly located posteriorly, in the inferior parietal areas during the first time window, evolving with time from the inferior parietal lobule to the superior parietal lobule.

The overall brain activity of the two dimensions reveals neurofunctional differences between the estimation of quantity and the estimation of time:

(1) At the source level; and contrary to the processing of duration, the processing of quantity implies a role of the frontal lobe at least for the present task.
(2) Some weak temporal activity differences were also shown for quantity, but not for duration.

The results have also revealed commonalities across the dimensions:

(1) both dimensions involved activations of the occipital areas from a very early stage of processing, although an stronger effect for duration was observed; and
(2) both dimensions were associated with the use of the anterior cingulate, when approaching the stage of decision-making, likely reflecting differences in the activation of the executive processes when the action is required (Menon and Uddin, 2010).

Occipital sources were, in fact, found during the first windows of analysis from 80 to 500 ms, specifically, in the cuneus and the middle occipital cortex. This is not surprising, since the paradigms, utilized in this study, relied on the visual modality. The occipital effects were more prominent for duration rather than for numerosity. Given the flash repetition of the visual stimuli in the quantity task, such visual areas could have shown adaptation (Grill-Spector and Malach, 2001; Gilaie-Dotan et al., 2008), thus, explaining the observed weaker effects for that task. Alternatively, this could also be explained from the perspective of brain connectivity. If numerosity task entails topographic representation in the parietal cortex from the very early stage, the same may not be necessarily true for the duration task. Hence, the weight of the numerosity processing would be transferred early to the parietal cortex, but it could rely more on occipital and cingulate areas for the processing of duration. This, however, assumes that there are no topographic representations for time intervals of different duration, which is an empirical question.

Regarding the temporal lobe sources, underlying the processing of quantity, they are rarely reported in studies focused on numerosity estimation. Given the presentation speed rate of the stimulus, they are unlikely related to counting during our task. It is more likely that they reflect the use of individuation processes. That is, visual enumeration depends on the capacity to process multiple dots in series, albeit in a linear fashion (Pagano and Mazza, 2012; Cantrell and Smith, 2013). Moreover, this linear individuation of the dots implies a working memory and attentional components that would, in turn, explain the frontal differences in the observed activity (Hyde and Wood, 2011; Piazza et al., 2011). This individuation and encoding processes differed between dimensions, being absent for the estimation of duration.

### Numerosity and Duration Within the Parietal Lobe

Crucially, the focus of our source analyses on the parietal lobes revealed both overlap and dissociations between the two studied dimensions. The most prominent effect was observed in the inter-hemispheric differences: while estimation of numerosity relied similarly on both parietal lobes (left and right IPS and left precuneus, and also bilateral inferior parietal areas), the estimation of duration was unbalanced toward the right parietal lobe, with the exception of the left precuneus. Right hemisphere activations involved the right inferior parietal lobule, and the right superior parietal lobule, including right IPS. Consequently, the present data suggest that there are also different parietal networks for the processing of two dimensions. At the same time, they have *at least* some regions of overlap. This overlap was most prominent in the left precuneus, as well as in the right and left superior parietal lobes, accompanied by the right supramarginal gyrus.

First, the association between duration and numerosity estimation in the left precuneus was the largest overlap. Left precuneus effects occurred under almost all windows of analyses. Precuneus has been never explicitly proposed as a region for such overlap, even though there are studies, demonstrating activation of this area separately for time processing (Dušek et al., 2012) and quantity (Dehaene et al., 2003; Haist et al., 2015). In fact, activations of this area during number comparison have been previously reported and based on meta-analytic data (Dehaene et al., 2003). For duration processing, a recent neuroimaging evidence exists, pointing toward an association between deficient duration estimation and Parkinson’s disease in the precuneus. Precuneus, in turn, is a part of the cognitive network, involved in Parkinson disease. Furthermore, these patients have impairments in time processing that can be ameliorated with dopamine medication, targeting that particular network (Dušek et al., 2012). Precuneus shows abnormal activity during duration processing in these patients (Artieda et al., 1992; Riesen and Schnider, 2001; Dušek et al., 2012). Such hemisphere overlap in precuneus is in agreement with the data, demonstrating an involvement of left parietal areas in temporal processing (Coull and Nobre, 1998; Coull et al., 2000, 2004; Livesey et al., 2007), both in studies with patients (Coslett et al., 2009) and in healthy population (Handy et al., 2003).

Other sites of overlap, observed in our present study, were the intraparietal sulci. They are, sometimes, the main brain regions of interest, when targeting the parietal commonalities between the dimensions of duration and numerosity (Bueti and Walsh, 2009; Dormal et al., 2012a; Skagerlund et al., 2016). Overlap in the right intraparietal sulcus has been demonstrated by using stimulation techniques and neuroimaging (Alexander et al., 2005; Bueti and Walsh, 2009; Dormal et al., 2012a; Dormal and Pesenti, 2013; Hayashi et al., 2013b). The IPS has been reported as a key locus for essential numerical processing along the developmental span and species (Dehaene et al., 2003; Nieder and Dehaene, 2009). Hence, the present data is in agreement with the previous literature, and suggests an overlap in the posterior parietal cortex.

Finally, the right inferior parietal cortex is usually considered as one of the key components of the brain network, supporting the processing of temporal intervals (Lewis and Miall, 2003a; Wittmann, 2009; Bonato et al., 2012). The disruption of the inferior parietal cortex by transcranial magnetic stimulation (TMS) has been shown to alter temporal estimations (Hayashi et al., 2013a). Neuroimaging data has demonstrated activation in the inferior parietal cortex during the measurement of duration (Rao et al., 2001; Harrington et al., 2004). Moreover, lesion analyses of stroke patients with temporal perception deficits show the supramarginal gyrus as a common area of damage (Harrington et al., 1998; Danckert et al., 2007). The ultimate role of the inferior parietal areas has been related to sustained attention in time (Rubia and Smith, 2004). Neuroimaging studies have found activations for both number and time processing, although no complete overlap was observed (Skagerlund et al., 2016).

Our data, thus, demonstrate several parietal areas of overlap between the two studied dimensions. The final, yet crucial, question, raised by the present study, was whether the observed regions of overlap support the same functionality for processing of each dimension.

### Parietal Overlap but Functional Divergence

Intersection in key numerical areas (i.e., IPS) would favor a view of a common magnitude system for time and number, as explicitly suggested by the ATOM model (Walsh, 2003; Bueti and Walsh, 2009). However, our observed correlation between the described overlapping sites and behavior indicates that dissimilar parietal regions appear to be preferably linked to the behavioral measures for each dimension and, therefore, support different function for each of the studied domains. Right superior parietal cortex effects (IPS) are associated with RTs differences for the contrast between numerosities, whereas the left and right precuneus, together with the right supramarginal gyrus, appear to be associated with the behavioral outcome during the contrast of durations. These areas are, respectively, considered as key parts of the numerical and the time processing networks.

Therefore, while considering the overlaps in these areas, our data adds an essential part of information to the currently existing models of magnitude processing in humans. Indeed, it points toward the parietal overlap, yet with differences in the relative functional weight for each of the common sites: the right IPS should be fundamental for the contrast of numerosities, supported by other parietal areas, and the right SMG and precuneus should be fundamental for the contrast of durations, supported by a wider right hemisphere network. This, in turn, implies that a spatial parietal overlap does not necessarily mean an exactly equivalent parietal magnitude system supporting processing for each dimension. Our data is in agreement with more local approach, taken by Harvey et al. (2013, 2015). It is worth noting that the parietal site reported by these authors as showing topographic representations of numerosity [MNI(*SD*): 23(4), -60(7), 60(7)], and that differ in its mechanisms to other non-numerical magnitude, coincides with the here reported parietal site directly related with behavior in numerosity (MNI: 24, -62, 60). Here we speculate about the possibility that such different neural mechanisms might have emerged as a consequence of the interaction between different brain networks, that might support other levels of processing for each dimension.

Our findings do not disregard the possibility that disrupting the areas of overlap via targeted stimulation would lead to behavioral failure for both dimensions, hence due to disruption of interdependent nodes within distinct networks. For the same reason, the present data does not disregard either that the processing of the two dimensions could interact at some point. Further research should explore the actual functional connectivity between the relevant sites detected in the present study, which might strengthen the conclusions presented here regarding the functional divergence of overlapped parietal areas.

## Data Availability

The raw data, supporting the conclusions of this manuscript, will be made available by the authors, without undue reservation, to any qualified researcher, contingent upon granted permission by Office of Sponsored Programs (OSP) at Florida Hospital (Advent Health).

## Author Contributions

ES designed the research, analyzed the data, and wrote the manuscript. MK and EC supervised the data collection and wrote the manuscript. TK, SM, and AV performed the data collection.

## Conflict of Interest Statement

The authors declare that the research was conducted in the absence of any commercial or financial relationships that could be construed as a potential conflict of interest.

## Footnotes

1 http://neuroimage.usc.edu/brainstorm
